# Ammonia stimulates growth and nitrite-oxidizing activity of *Nitrobacter winogradskyi*

**DOI:** 10.1080/13102818.2014.901679

**Published:** 2014-05-08

**Authors:** Shouguang Ma, Demin Zhang, Wenjun Zhang, Yinong Wang

**Affiliations:** ^a^ Key Laboratory of Applied Marine Biotechnology of Ministry of Education, Ningbo University, Ningbo, P. R. China

**Keywords:** *Nitrobacter winogradskyi*, nitrite-oxidation, ammonium

## Abstract

The aim of this study was to obtain a nitrite-oxidizing bacterium with high nitrite oxidation activity for controlling nitrite levels. A nitrite-oxidizing bacterium, ZS-1, was isolated from the water of a coastal *Pseudosciaena crocea*-rearing pond. The strain was identified as *Nitrobacter winogradskyi* based on the phylogenetic analyses of the 16S ribosomal ribonucleic acid gene and *nxrA* sequence of ZS-1. Under aerobic condition, the nitrite-oxidizing activity of ZS-1 did not change considerably in the range of pH 7–9, but was strongly inhibited by lower (pH = 6) and higher (pH = 10) pH values. The optimum temperature range is 25–32 °C. Lower temperature made the adaptive phase of ZS-1 longer but did not affect its maximum nitrite oxidization rate. The nitrite-oxidizing activity of ZS-1 started to be inhibited by ammonia and nitrate when the concentrations of ammonia and nitrate reached 25 mg L^−1^ and 100 mg L^−1^, respectively. The inhibition was stronger with higher concentration of ammonia or nitrate. The nitrite-oxidizing activity of ZS-1, however, was not inhibited by high concentration of nitrite (500 mg L^−1^). The nitrite-oxidizing activity of ZS-1 was increased by low ammonia concentration (1 mg L^−1^ to 10 mg L^−1^).

## Introduction

Nitrification is an important step of the global nitrogen cycle. Through biological oxidation, ammonia is oxidized to nitrite and further to nitrate. Nitrification is catalysed by two types of reactions. The first type of reaction is the oxidation of ammonia to nitrite by ammonium-oxidizing bacteria (AOB) or ammonia-oxidizing archaea (AOA). The second type of reaction involves the oxidation of nitrite to nitrate by nitrite-oxidizing bacteria (NOB, also known as nitrifying bacteria).[1] NOB can be divided into five groups: *Nitrobacter*, *Nitrococcus*, *Nitrospina*, *Nitrospira* and the newly discovered *Candidatus Nitrotoga*. Based on the 16S ribosomal DNA (rDNA) phylogenetic analysis results, *Nitrobacter*, “*Candidatus Nitrotoga*”, *Nitrococcus* and *Nitrospina* belong to α, β, γ and δ classes of Proteobacteria, respectively. *Nitrospira* belongs to phylum *Nitrospira*.[2,3]

In recent years, aquaculture production is increasing. However, water is deteriorated and the disease occurrence rate is increasing. The main reason for water quality deterioration is the elevated nitrite concentration.[4,5] In freshwater, the nitrite concentration is increased up to 1 mg/L, and in sea water it is increased to 20 mg/L. Nitrite is toxic to organisms.[6] One of the effective measures to reduce nitrite level is addition of nitrifying bacteria to water,[7,8] thus increasing the efficiency of nitrite oxidation. Therefore, it is important to screen and isolate NOB. In this study, several strains of NOB were enriched and isolated. Strain ZS-1 with efficient nitrite oxidation activity was selected to investigate the environmental effects on the removal of nitrite.

## Materials and methods

### Bacteria and growth conditions

The water sample was collected from the coastal *Pseudosciaena crocea* rearing pond. In this study, 10 mL of the sample was dispensed into 250 mL of Erlenmeyer flasks containing 100 mL of *Nitrobacteria* enrichment medium (NaNO_2_ 0.2 g, Na_2_CO_3_ 1 g, NaC1 0.5 g, K_2_HPO_4_ 0.5 g, MgSO_4_·7H_2_O 0.5 g, FeSO_4_·7 H_2_O 0.4 g, ddH_2_O 1000 mL, pH 7.8), with shaking at 30 °C. More NaNO_2_ would be added once the culture mixture displayed achromaticity as detected by using the Griess solution method. After the fourth portion of NaNO_2_ added, 10 mL of culture was transferred to a new enrichment medium and incubated in the dark for 4 months with a blank control. The enrichment mixture was serially diluted and spread over a plate with agarose instead of agar (13 g L^−1^ in enrichment medium). After a 15 d incubation at 30 °C, single needle-like colonies were isolated and pure culture was obtained by streaking on the new isolation medium (NaNO_2_ 0.2 g, CaCl_2_·2H_2_O 0.05 g, MgSO_4_·7H_2_O 0.1 g, FeSO_4_·7H_2_O 0.001 g, KH_2_PO_4_ 0.0017 g, CuSO_4_·5H_2_O 6 mg, Na_2_MoO_4_·2H_2_O 25 mg, MnCl_2_·4H_2_O 50 mg, CoCl_2_·6H_2_O 0.5 mg, ZnSO_4_·7H_2_O 25 mg, H_2_O 1000 mL, pH 6.5–7.0). NOB were grown in pure cultures on isolation medium containing 100 mg L^−1^ 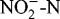 at 30 °C, with 150 r min^−1^ shaking. Stock cultures for the strain with the highest nitrification efficiency were maintained at 17 °C in the dark.

### Morphological observation

Morphological examinations were performed with the pure culture plates. To characterize the strain, microscopic observations and measurements were carried out.

### PCR

Total genomic DNA was extracted from ZS-1 and used for the template of polymerase chain reaction (PCR) amplification. For amplification of the 16S rDNA region, primers 27f (5′-AGAGTTTGATCMTGGCTCAG-3′) and 907r (5′-CCGTCAATTCMTTTRAGTTT-3′) were used and the PCR amplification was conducted as described in Alawi et al.[9] PCR products of the predicted length were visualized by electrophoresis on a 1.0% agarose gel and purified with the SanPrep gel extraction kit (Sangon Biotech, Shanghai, China) following the manufacturer's instructions. The purified fragments were cloned into the pMD-19 T simple vectors and transformed into TG1 *Escherichia coli* cells. Recombinants were selected by blue/white screening, according to the procedure described by Sambrook et al.[10] The positive clones were further confirmed by PCR.

Amplification of *nxrA* was carried out as described by Poly et al. [11] with a forward primer F1370 F1 *nxrA* (5′-CAGACCGACGTGTGCGAAAG-3′) and a reverse primer F2843 R2 *nxrA* (5′-TCCACAAGGAACGGAAGGTC-3′). All primers were synthesized by Invitrogen (Shanghai, China) and the produced amplicons were sequenced by Shanghai Sangon Biotech Co., Ltd.

### Data analysis

Sequence similarity search of the 16S rDNA and *nxrA* fragments obtained from the PCR assay was performed using Blastn against the NCBI nucleotide database (http://www.ncbi.nlm.nih.gov/BLAST/). Neighbour-Joining (NJ) analyses were performed using Mega 4.0 software with the parameter set to 1000 bootstraps.

### Nitrification activity analyses

Nitrification experiments were performed in 300 mL Erlenmeyer flasks containing 100 mL of medium. The starting cultivation conditions for ZS-1 were as follows: 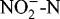 concentration, 20 mg L^−1^; culture temperature, 30 °C; and the shaker speed, 150 r min^−1^. Three independent tests were performed for all experiments.

To characterize the nitrification activity of the nitrite-oxidizing strain ZS-1, the effects of pH, temperature and three forms of nitrogen on ZS-1 were investigated. The pH values included 6, 6.5, 7, 8, 9 and 10, with the liquid medium at pH 7 as the control. The temperatures were set at 10, 16, 20, 25, 28, 30, 32 and 37 °C, respectively. Nitrification activity of ZS-1 at each temperature was measured after incubation of the bacterium in 10 mg L^−1^ 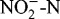 in the dark without shaking. To assay the effects of the different forms of nitrogen, experiments at various concentrations of NH_4_^+^-N (0, 1, 10, 25, 50, 100, 200 and 300 mg L^−1^), 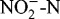 (1, 10, 50, 100, 200, 400 and 500 mg L^−1^), and 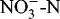 (0, 1, 10, 50, 100, 200, 400 and 500 mg L^−1^) were conducted.

The 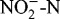 and 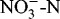 were detected using the Diazo-azo method and ultraviolet spectrophotometric method, respectively. The culture growth was monitored by measuring the OD_600_ values. Specific bacterium populations were detected by the most-probable-number (MPN) method.

## Results and discussion

### A strain of bacterium with high nitrite oxidation rate is identified

**Figure 1.**
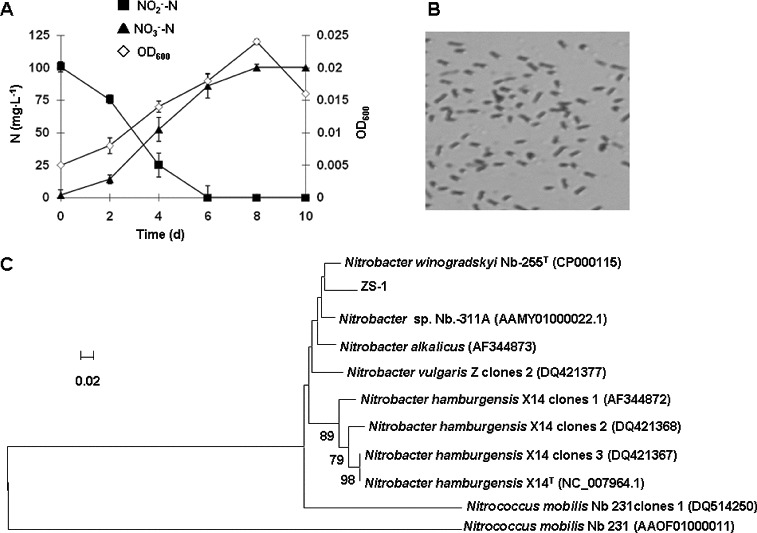
Bacterial strain ZS-1 with high nitrite oxidation rate. Oxidization of 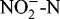 by ZS-1 (A); concentration of nitrite-N (▪) and nitrate-N (▴); OD_600_ (◊). Light microscopic photograph of ZS-1 (B); ×1000 magnification; dyed with crystal violet. Phylogenetic tree (C) generated based on the partial sequence of the *nxrA* gene of ZS-1; neighbour-joining algorithm; nodes supported by bootstrap values (only values >60 are shown); scale bar, 2% sequence divergence.

To obtain NOB with high nitrite activity, bacteria were isolated from a *P. crocea*-rearing pond and the nitrite oxidation rate was studied. Three strains of NOB were isolated from the water sample collected from the coastal *P. crocea*-rearing pond. Only strain ZS-1, which had the highest nitrite oxidation rate among the three strains, was chosen for further study. All the nitrite substrate was converted to nitrate by ZS-1 within six days, reaching a highest OD_600_ value of 0.024 and a biggest cell yield of 6.67 × 10^6^ during the whole nitrite-oxidizing process (Figure 1(A)).

### ZS-1 is identified to be *Nitrobacter winogradskyi*

Colonies of ZS-1 were needlepoint-sized, light brown, round, and smooth edged. The bacterium was short rod-shaped, (0.4–0.49) μm wide and (1.09–1.54) μm long, gram-negative (Figure 1(B)). To identify the molecular characteristics of ZS-1, we performed PCR using the 16S rDNA and *nxrA* genes as templates. An amplicon of 873 bp was obtained by PCR using the 27f/907r primer pair for 16S rDNA. The PCR with F1370 F1 *nxrA*/F2843 R2 *nxrA* primer pair generated a 320 bp fragment from *nxrA*. Using the Blastn software based on the GenBank nucleotide database, the sequence comparison results indicated that the 16S rDNA was 100% similar to the corresponding sequence in *Nitrobacter winogradskyi* NB-255^T^ (GenBank Accession CP000115). The *nxrA* sequence was 98% similar to the corresponding region of the *Nitrobacter winogradskyi* NB-255^T^. The constructed NJ tree based on the functional gene *nxrA* is shown in Figure 1(C). The *nxrA* phylogenetic result, together with the 16S rDNA sequence analysis results, suggests that ZS-1 is *N. winogradskyi*.

### ZS-1 has an optimum pH range of 7–9

**Figure 2.**
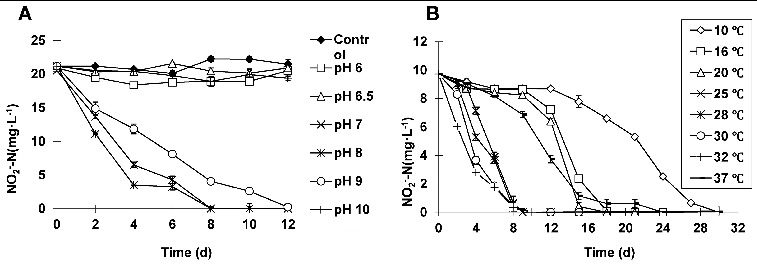
Optimum pH range and temperature adaptation of ZS-1. Effect of the initial pH value on nitrite oxidation by ZS-1 (A). Effect of temperature on nitrite oxidation by ZS-1 (B).

To study the effect of pH value on nitrite oxidation by ZS-1, nitrite oxidation was determined at various pH conditions. The results indicated that when the initial pH value was set at 6, 6.5 or 10, there was no considerable difference in 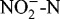 changes compared to the control, suggesting complete suppression of the nitrification activity of ZS-1 (Figure 2(A)). When the initial pH values were 7 or 8, similar results were obtained, with higher nitrification efficiency at pH = 8. Approximately 50% and 80% of the 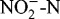 were removed by nitrification on day 2 and day 4, respectively. All 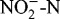 was removed as detected on day 8. At an initial pH value of 9, 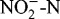 with a concentration of 20 mg L^−1^ was completely oxidized on day 12, showing a decrease in the nitrification activity. The experiments were repeated for more than 3 times, with similar nitrification results obtained. Thus, these results suggest that ZS-1 functions in a wide range of initial pH values, with an optimum pH range of 7–9.

The pH value is an important factor for nitrite oxidation, since it affects the nitrification process either by directly influencing the bacterium growth or by indirect involvement in the ionization of NO_2_/HNO_2_ or NH_4_^+^/NH_3_.[12] The optimal pH value is 7.9 for *Nitrobacter*,[13] with a decrease of 80% at pH 6 or 9. However, the oxygen uptake rate analysis showed that NOB was strongly suppressed at pH 6.5, while the nitrification activity was the same in the range of 7.5–9.95. For ZS-1, the pH value at which complete inhibition occurred was below 6.5 or above 10, without any activity changes at the pH range of 7–9, which is consistent with the results reported previously.[12]

### Good adaptation to lower temperature is shown for ZS-1 in nitrite oxidation

To study the effect of temperature on nitrite oxidation by ZS-1, nitrite oxidation was measured under several temperatures. Over the temperature range of 25–32 °C, nitrification activity was indicated to be increase in a temperature-dependent manner (Figure 2(B)). At 32 °C, ZS-1 showed the highest nitrification activity, decreasing the initial 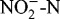 from 10 to 6.02 mg L^−1^ on day 2, and to 2.79 mg L^−1^ on day 4. At 16 and 20 °C, a slightly increased nitrification rate (0.067–0.083 mg L^−1^ h^−1^) was detected on 12–15 d after an adaptive phase (6–9 d). Over the temperature range of 25–30 °C, the nitrification rate varied between 0.067 and 0.096 mg L^−1^ h^−1^ within day 2–4. At the lowest temperature (10 °C), the nitrification process occurred with a relatively longer adaptive phase (12 d).

Temperature is one of the most important factors affecting the nitrification activity and population structure of NOB. The activity of *Nitrobacter* is optimal at the temperature range of 25–28 °C,[14–17] while the optimal conditions for ZS-1 are around 25–32 °C. Notably, the effect of low temperatures (such as16 and 20 °C) on nitrification was represented in an adaptive model. After an adaptive phase, the maximum nitrification rate range (0.067–0.083 mg L^−1^ h^−1^) appeared to be close to that at the optimal temperature range (0.067–0.097 mg L^−1^ h^−1^). However, the activity of the NOB showed a difference up to 1.5 times at the above two temperatures.[13,18] Thus, it was concluded that strain ZS-1 exhibited a better adaptation of lower temperatures, suggesting a wider application range for ZS-1.

### Nitrite oxidation activity of ZS-1 is promoted by a low concentration of ammonium

**Figure 3.**
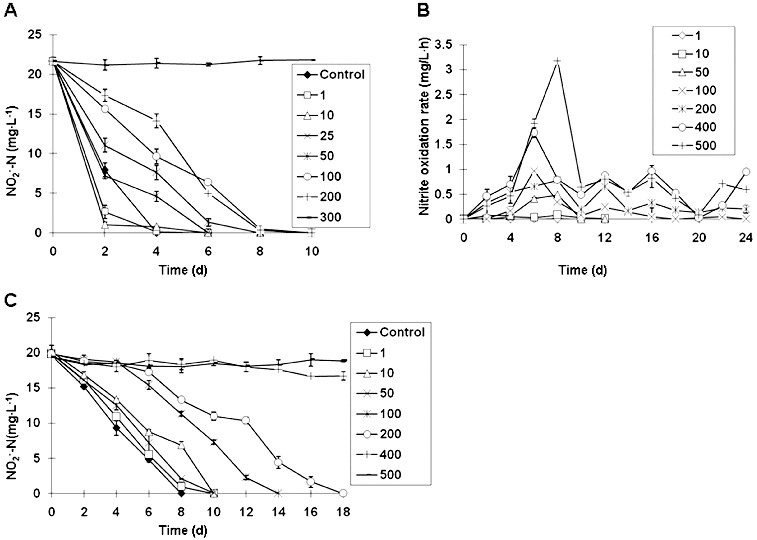
Nitrite oxidation activity. Effect of ammonia nitrogen (1–300 mg L^−1^) on nitrite oxidation by ZS-1 (A). Effect of nitrite concentration (1–500 mg L^−1^) on the average (▪) and the maximum (▴) nitrite oxidization rate by ZS-1 (B). Effect of nitrate concentration (1–500 mg L^−1^) on nitrite nitrification by ZS-1 (C).

To study the effects of ammonia nitrogen, nitrite nitrogen, nitrate nitrogen on oxidation of nitrite by ZS-1, nitrite removal was detected in the presence of various concentrations of ammonium. The increase in NH_4_^+^-N concentration over 25 mg L^−1^ triggered low nitrification effects, and the nitrification activity was almost decreased to 0 when the concentration reached 300 mg L^−1^ (Figure 3(A)). At lower concentrations (<25 mg L^−1^), such as 1 and 10 mg L^−1^, a slight increase in nitrification was observed in comparison to the control without NH_4_^+^-N. On day 2, the 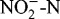 removal rates at 1 mg L^−1^ (87%) and 10 mg L^−1^ (95%) were 24% and 32% higher than that in the control (63%), respectively (*P* < 0.01). All repeated experiments showed that low NH_4_^+^-N concentration resulted in increases in nitrification rates.

NH_4_^+^-N influenced nitrification by acting as a nitrification inhibitor.[19,20] High concentrations of NH_4_^+^-N inhibited nitrite oxidation.[21] However, the inhibitory effect was due to the free ammonia (FA), rather than to NH_4_^+^-N.[22] Many studies on FA inhibition have been conducted, and the lowest FA concentrations for the complete inhibition in *Nitrobacter* are summarized in Table 1. In the present study, when the initial concentration of NH_4_^+^-N was 300 mg L^−1^, complete inhibition was obtained for ZS-1, and the FA was subsequently calculated as 74.16 mg L^−1^,[22] which is 7–70 fold higher than the data given in Table 1. Interestingly, low concentrations of NH_4_^+^-N (1–10 mg L^−1^, equal to FA at 0.25–2.47 mg L^−1^) led to an increase in the nitrification rate. To the best of our knowledge, this study is the first report on nitrification activity improvement caused by low concentration of NH_4_^+^-N. Considering that the NH_4_^+^-N concentration in the culture environment is mainly below 10 mg L^−1^, ZS-1 seems to be appropriate for nitrite treatment in waste water.

**Table 1.** The lowest concentrations of free ammonia (FA) for completely inhibition of nitrite-oxidizing by *Nitrobacter*.

FA (mg L^−1^)	Reference
1	[22]
8.9	[21]
1.5	[23]
10	[24]
6	[25]
10	[26]
74.16	This study

The peak nitrification rate was observed on day 6–8 at different 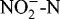 concentrations (Figure 3(B)). The average rate of oxidation at 50 mg L^−1^ was significantly higher than those at 1 and 10 mg L^−1^. At concentrations of between 100 and 500 mg L^−1^, the average nitrification rate was 0.20 mg L^−1^ h^−1^, 0.38 mg L^−1^ h^−1^, 0.67 mg L^−1^ h^−1^ and 0.89 mg L^−1^ h^−1^, respectively. The maximum rate, 3.18 mg L^−1^ h^−1^, was found at 500 mg L^−1^, while the nitrification rate at lower concentrations was found to be below the maximum value of 0.1 mg L^−1^ h^−1^. Compared to the control, there was no considerable difference in nitrification activity at low 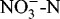 concentration (below 50 mg L^−1^). However, when 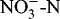 exceeded 100 mg L^−1^, nitrification was clearly inhibited, particularly at a concentration of 400 mg L^−1^, showing a strong inhibitory effect (Figure 3(C)).

The inhibition of substrate and product on *Nitrobacter agilis* was analysed with a bioreactor.[27] The result showed that all 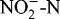 in the bioreactor was converted to 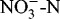 at a substrate concentration of 5600 mg L^−1^. Moreover, within the concentration of 100–1500 mg L^−1^, there was no effect on NOB nitrification.[13] Similarly, the nitrification rate of ZS-1 was increased by the increase of 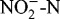 concentration (100–500 mg L^−1^), reaching the highest level (3.18 mg L^−1^ h^−1^) at 500 mg L^−1^.

The studies on oxidation kinetics of nitrite in *Nitrobacter winogradskyi* indicated a non-competitive inhibition model for 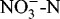.[28] For ZS-1, no inhibition was found below 50 mg L^−1^ of 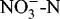, and the inhibition effect began to be detectable at 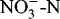 concentration of 100 mg L^−1^. Complete inhibition on ZS-1 was observed only at 400 mg L^−1^ of 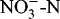. The results here suggest that ZS-1 developed strong tolerance to 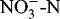.

## Conclusions

ZS-1, a NOB strain with high nitrification efficiency, was isolated and characterized in this study. Complete nitrification can be achieved in a wide range of temperatures and pH values, with a promotion effect of ammonia nitrogen. These characteristics are of an important value for application of ZS-1 in aquaculture water purification.
